# Using the Research Domain Criteria as a framework to integrate psychophysiological findings into stress management and psychotherapy interventions

**DOI:** 10.3389/fnrgo.2023.1245946

**Published:** 2023-09-29

**Authors:** Patrick R. Steffen

**Affiliations:** Department of Psychology, Brigham Young University, Provo, UT, United States

**Keywords:** stress, Research Domain Criteria, interventions, assessment, biofeedback, mindfulness

## Abstract

Research on the psychophysiology of stress is expanding rapidly, but the field lacks a clear integrative framework to help translate research findings into empirically supported stress interventions. The Research Domain Criteria (RDoC) is an excellent candidate to explore as a framework to integrate stress research. The RDoC framework is a dimensional, multi-modal approach to psychopathology proposed as an alternative to categorical approaches used by the International Classification of Diseases (ICD) and the Diagnostic and Statistical Manual (DSM). The goal of this paper is to explore the RDoC as a framework to integrate psychophysiology research into therapeutic interventions for stress. The RDoC consists of six domains: negative valence systems, positive valence systems, cognitive systems, social processes systems, arousal/regulatory systems, and sensorimotor systems, and provides an excellent structure for integrating information from multiple levels of functioning including physiology, behavior, and self-report, as well as genes, molecules, cells, and brain circuits. Integrating psychophysiological research on stress using the RDoC framework can direct and amplify stress management and psychotherapeutic interventions. First, the RDoC provides a clear foundation for conceptualizing the stress response in terms of important concepts such as allostasis and adaptation. In this perspective, the terms “allostatic response” or “adaptation response” are more descriptive terms than “stress response” in understanding bodily responses to life threats and challenges. Second, psychophysiological approaches can be used in the context of modalities such as biofeedback and mindfulness to both collect psychophysiological data and then integrate that data into a broader therapeutic framework. Heart rate variability (HRV) biofeedback is being used more frequently as part of a therapeutic intervention package with stress management and psychotherapy, and HRV data is also used to provide outcome evidence on the efficacy of treatment. Mindfulness practices are commonly used in combination with stress management and psychotherapy, and psychophysiological data (HRV, EEG, blood pressure, etc.) is often collected to explore and understand mind/body relationships. In conclusion, the lack of a clear framework to assess and understand mind/body functioning limits current stress research and interventions. The RDoC provides a strong framework to assess and integrate physiological and psychological data and improve stress interventions.

## Introduction

Stress significantly impacts health and wellbeing and national stress surveys indicate that stress remains at high levels following the pandemic (Schneiderman et al., 2005; Cohen et al., 2007; American Psychological Association, 2023). Therefore, effective stress interventions are of vital importance. Although there is a large and growing body of psychophysiological research on stress, the field lacks an accepted overarching framework to integrate psychophysiological stress research findings into stress interventions. The biopsychosocial model is frequently used as a general model to conceptualize the interactions between biological, psychological, and social factors in health (Searight, 2016; Smith, 2021). The biopsychosocial approach, however, has focused only on general concepts and has not provided a specific framework for integrating information across factors. Cohen et al. (2016) note that there are multiple traditions in studying stress, from epidemiological to psychological and biological, with each using different methods to study stress and even have different conceptualizations of stress. Definitions of stress used by researchers, as well as by people who are not specialists, are often vague and lack clarity, with some experts arguing that the word stress should not used at all (Ader, 1980; Koolhaas et al., 2011; Kagan, 2016).

The Research Domain Criteria (RDoC) approach provides a promising framework within which to conceptualize stress and integrate current stress research findings into stress interventions (Craske, 2012; Cuthbert and Kozak, 2013; Kozak and Cuthbert, 2016). The RDoC consists of six domains (negative and positive valence systems, cognitive systems, social processes systems, arousal systems, and sensorimotor systems) that are impacted by environment and development and are studied using a multi-modal approach, from genes to broad social factors (Vaidyanathan et al., 2020). Having a clearly delineated framework can facilitate the integration of research into interventions by mapping out specifically the domains to cover, environmental and developmental factors that impact these domains, and the multiple dimensions to consider in a comprehensive stress assessment. This paper begins by reviewing how stress has been defined and understood over the years, the RDoC approach is then presented, and then an integrative method is considered using HRV biofeedback and mindfulness as specific approaches to guide both assessment and interventions.

### Understanding stress

In the 1950′s, Selye's extensive work on stress significantly impacted how the word began to be used in everyday language (Selye, 1956; Lazarus, 1993; Becker, 2013). Up until at least 1949, dictionaries defined stress only as the action of external forces (e.g., a natural disaster) that people endured, or the physical strain placed on an object such as a bridge. Selye's research made it clear that stress has significant internal physiological impacts, and that stress is an internal event as well as an external one. Problems arose, however, with the vagueness of the word stress, with one critic noting that “therefore stress, in addition to being itself and the result of itself, is also the cause of itself” (Roberts, 1950). To define the concept of stress more clearly, Selye (1976) defined stressor as a cause of stress and emphasized the terms distress and eustress to help delineate negative and positive forms of stress. Selye was not a native English speaker and later wished he had used another term besides the word stress to describe the body's response to life challenges.

Today it is still argued that the word stress is too ambiguous to be useful, being more of an impediment to research than a help (Ader, 1980; Koolhaas et al., 2011; Kagan, 2016). Most researchers, however, do continue to use the word stress, with stress researchers arguing that the remedy to this problem is increased specificity in how the word stress is used to maximize clarity in what is meant, and broader conceptual models that integrate biological, psychological, sociological, and epidemiological approaches (Cohen et al., 2016; Slavich and Shields, 2018; Croswell and Lockwood, 2020; Croswell et al., 2022). For example, to help clarify stress terms, instead of just using the word stress, use the word stressor to define the event and chronic stress to help clarify time period.

Given that researchers struggle with word “stress”, it is not surprising that lay people who do not have specialized knowledge about stress have difficulties understanding as well. Lay people still typically emphasize stress as a negative event or experience; even the Oxford dictionary defines stress using primarily negative words. This is in spite of repeated attempts by researchers and professionals to have a broader definition of stress, emphasizing that stress can have positive as well as negative effects. Selye (1976) emphasized positive stress with his concept of eustress. Hanson (1986) book, “The Joy of Stress,” emphasized the positive side of stress and was well-received and lauded. And in 2013, McGonigle (2013) presented a famous TED talk about making stress our friend, emphasizing the positive side of stress. None of these have seemed to make a dent in the overall negative view of stress. Given the confusion over the use of the word stress, when working with patients and people it may be more helpful to focus on the process of coping with life challenges and use more descriptive terms instead of using the word stress.

Perhaps most importantly, the concept of stress is really a metaphor that incompletely addresses the physiological and psychological processes that occur during challenge and threat (Thibodeau and Boroditsky, 2011; Taylor and Dewsbury, 2018). The problem with metaphors being taken literally is that no metaphor completely maps onto reality. People are not bridges made of concrete and steel withstanding physical strain; people are biological, psychological, and social organisms that grow, adapt, and change over time (Lazarus, 1993; Thibodeau and Boroditsky, 2011; Taylor and Dewsbury, 2018). People can be negatively impacted by chronic stress over time, but the process is psychological and social as well as biological. To make things even more complicated, two people can perceive the exact same event in diametrically opposite ways. As a simple example, some people love rollercoasters and some people fear them, with the same experience leading to very different physiological responses. Bridges do not perceive, respond, or grow as humans do. This is where taking the metaphor of stress too literally causes problems, as many people conceptualize stress as something that happens to them as if they were merely an object being acted upon, not recognizing that their perception of the event plays a key role in the stress response. Interestingly, perception is typically a key focus of stress interventions (Pretzer and Beck, 2007).

Modern views of stress emphasize challenge or threat to homeostasis, the person's perception of stress, and their perceived ability to cope (McEwen, 2000; Goldstein and Kopin, 2007; McEwen and Akil, 2020). The process of maintaining balance between internal needs and external demands is at the heart of the stress response. A person's perception of their ability to cope with life challenges directly impacts this balance. This process is well-described by the concept of allostasis (McEwen, 2000). Allostasis, or maintaining stability through change, is a broader term than homeostasis focusing on interdependence of bodily systems in response to life's challenges (McEwen, 2000). Bodily systems need to work together as an integrated whole to effectively deal with life challenges. Allostatic load, or the cost of chronic exposure to significant life challenges, can negatively impact system functioning over time leading to increased morbidity and early mortality (McEwen and Akil, 2020). Selye (1946) concept of “diseases of adaptation” also maps well with the ideas of allostasis and allostatic load. Maintaining healthy allostasis is basically healthy adaptation. Instead of using the term “stress response” with people, the terms “allostatic response” or “adaptation response” are both more accurate and more descriptive of what people experience during life challenges (McEwen, 2000) (see Table 1).

**Table 1:** Definitions of stress.

**Concept**	**Definition**
Stress	The perceived demands of a situation exceed or threaten to exceed the perceived resources of the individual. Selye conceptualized stress as the effects of anything that threatens homeostasis.
Stressor	Situations or events that are perceived as threatening or challenging.
Stress response	Attempts to cope with and reduce stress. There are significant individual differences in how people perceive and respond to stressful situations.
Eustress	Positive stress
Allostasis	Maintaining stability through change. The major goal of allostasis is to promote adaptation.
Allostatic load	The cumulative burden of chronic exposure to stress that may cause physiological dysregulation and promote disease.
Adaptation response	The ability to cope with external environmental conditions while maintaining necessary internal functioning required for life (oxygen, glucose, etc.)

Psychophysiological research emphasizes the importance of balance and coping (Lang et al., 1990, 2017; McEwen, 2000; McEwen and Akil, 2020), and an understanding of psychophysiological responses to life challenges provides a strong foundation for stress interventions. The brain integrates information about life challenges and decides on appropriate action, and the endocrine and peripheral nervous systems provide communication highways throughout the body preparing the body for that action. Cortisol and adrenaline (epinephrine) are key chemical messengers of these two respective systems. Although they are often called “stress hormones”, it is more helpful to think of them as “hormones involved in the stress response” because their primary purpose is to assist in metabolism and energy regulation regardless of stress levels. They are more than just stress hormones, they help regulate normal, day to day activities. Given that energy utilization is often a key part of the stress response, cortisol and adrenaline also play a central role in the stress response as well. Excessively high levels of cortisol and adrenaline over time are associated with dysregulation of the cardiovascular, immune, and gastrointestinal systems, as well as with the central nervous system itself. An effective framework for understanding stress and conducting stress interventions needs to consider the different organ systems involved in stress, the multiple physiological levels in stress (i.e., chemical messengers, cells, brain circuits, organs and organ systems), the environmental and social contexts in which they occur, and how all of these interact together over time in processes of development and change.

## The Research Domain Criteria as an integrative framework for stress

The RDoC approach provides an excellent framework for integrating psychophysiological research into psychotherapy and stress management interventions (Craske, 2012; Cuthbert and Kozak, 2013; Kozak and Cuthbert, 2016; Vaidyanathan et al., 2020). Although definitions of stress are becoming clearer and assessments of stress are becoming more refined, stress research lacks an agreed upon overarching framework for integrating knowledge across theory, assessment, and intervention, and providing a clear way to translate research into practice. In particular, stress interventions often focus on the symptoms without considering the underlying causes. Having a clear, organized framework that integrates research knowledge across theory, assessment, and intervention has the potential to improve how stress interventions are conducted to improve allostasis and adaptation. There are other frameworks being developed in addition to the RDoC that may be useful for integrating research, particularly the Hierarchical Topology of Psychopathology (HiTOP), and Michelini et al. (2021) provide an interesting integration of RDoC and HiTOP approaches. For this article the focus is exclusively on the RDoC as a beginning point to explore possibilities.

The RDoC approach provides an excellent framework for integrating stress research and stress interventions for four key reasons. First, the RDoC takes a dimensional approach being created as a dimensional alternative to the DSM categorical approach. Depressive symptoms, for example, occur along a continuum with many possible levels; to categorize people as either depressed or not depressed is to lose vital information. Similarly, stress is not an either/or proposition with people either having a lot of stress or none at all. Stress also occurs along a continuum from low to high with many possible levels. Second, the RDoC takes current environments into account and focuses on the development of distress over time. The current stress context is not independent of previous developmental factors, and assessing these factors may have significant benefit for interventions. Third, the RDoC takes a multilevel assessment approach, ranging from self-report and interviews to assessing organ systems, brain circuits, cells, and chemical messengers. Taking a broad, integrative multilevel approach to assessment enables a thorough representation of stress to be constructed. Fourth, the RDoC is a developing framework, continually integrating new research discoveries over time. The seven pillars of the RDoC approach (e.g., the RDoC is flexible and dynamic to accommodate research advances, focuses on constructs with solid evidence, and uses reliable and valid measures) emphasize this approach to continual development (see Table 2).

**Table 2:** The seven pillars and six domains of the Research Domain Criteria.

**Pillar**	**Definition**
Translational perspective	Understanding based on basic science, including basic systems such as neural, behavioral, cognitive, etc.
Dimensional approach	Constructs are dimensional and not categorical
Reliable, valid measures	Constructs assessed using measures with demonstrated reliability and validity
Novel research designs	New research designs and strategies are needed to aid in new discoveries
Integrative methods	Emphasis on integrating multiple types of measures to better study constructs
Platform for research	Goal is to have a platform for research based on solid evidence, and not tied to any one approach
Flexible, dynamic to accommodate research advances	Help free researchers from constraints of any given diagnostic system to help push understanding forward
**Domain**	**Definition**
Negative valence	Responses to threat or harm through self-protection and avoidance
Positive valence	Responsiveness to rewards and approach behavior
Cognitive	Attention, perception, memory, and effortful cognitive control
Social	Affiliation and attachment, social communication, self-perception and understanding, and other-perception and understanding
Arousal	Arousal, circadian rhythms, sleep-wakefulness
Sensorimotor	Control and execution of motor behaviors, agency and ownership, habit, innate motor patterns

The RDoC dimensional, developmental/environmental, and multilevel assessment approach is measured across six domains: negative valence systems, positive valence systems, cognitive systems, social systems, arousal systems, and sensorimotor systems. Each of these domains will be discussed in turn.

### RDoC negative and positive valence systems

Avoidance of harm and approaching needed resources are key to survival. From a psychophysiological perspective, we are guided by the two core motives of approach and avoidance, with affect guiding our strategic orientation toward the world (Lang et al., 1990, 2017). Our current valence, the balance between our current negative and positive affect, provides a background framework for the interactions between ourselves and our various environments. This is also termed core affect which represents the integration of information indicating how we are doing each moment in time (Russell, 2003; Duncan and Barrett, 2007).

Negative valence systems direct our responses to threat or harm through self-protection and avoidance. To survive and thrive, negative valence systems help us to effectively cope with life threats and challenges as they occur. LeDoux (2012, 2021) argues that we do not have fear circuits, rather we have fast acting survival circuits that motivate and guide behavior. Fear, on the other hand, is the conscious awareness of our survival response. The goal of negative valence systems is to be vigilant and ready to respond quickly to danger. How can the negative valence systems go wrong during chronic stress? Because safety and survival are paramount, human beings have a natural tendency to err on the side of protection, with the stress response being the default response until safety is certain (Brosschot et al., 2018; Van den Bergh et al., 2021). If a significant threat is missed, the results could be disastrous. If life is chronically uncertain, then the stress response becomes chronic and continually engaged to be ready just in case, even when no stressors actually occur. From this perspective prolonged stress responses can occur in common situations, situations in which no current stressors are occurring, such as loneliness, low social status, or early life adversity. Therefore, a person does not need to be currently experiencing stress for a stress response to occur, merely feeling unsafe is enough to trigger a stress response (Brosschot et al., 2018). In terms of stress interventions, the question then is not what causes a prolonged stressor but what stops it, what contributes to a sense of safety?

The positive valence systems consist of responsiveness to rewards and approach behavior. To survive we need to be able to obtain needed resources such as food. How can the positive valence systems go wrong during chronic stress? The positive valence systems can go wrong as people approach substances or activities that can have a negative impact, such as using addictive substances or chronic gambling. Initially, these activities can lead to positive feelings and increased desire to continue using. Over time, they can lead to addiction as people engage in these activities to increase their sense of positivity. Eventually, tolerance develops as more and more use is required to obtain positive feelings, and use begins to be about avoiding the negative feelings of withdrawal.

### RDoC cognitive and social systems

As with the negative and positive valence systems, the central goal of the cognitive and social systems is survival. According to Bowlby's theory of “environment of evolutionary adaptedness”, humans' successful evolution over hundreds of thousands of years was based on increased cognitive abilities and strong social connections (Bowlby, 1980). We have cognitive processes such as problem solving, planning, effective decision making, etc., because they are highly adaptive. We have social relationships because there is strength and protection in numbers, we can accomplish more together than alone, and we need parents to make it out of infancy. When our cognitive and social systems are doing well, we are doing well.

The cognitive valence systems consist of attention, perception, memory, and effortful cognitive control. By attending to and being aware of our current situation we can more successfully respond to emerging needs. Our brains are geared to predict coming need to be ready for whatever changes, good or bad, might arise. How can cognitive systems go wrong with chronic stress? Our attention and perception are impacted by our motivation and affective state (Lang et al., 1990, 2017). The perseverative cognition hypothesis argues that worry and rumination are common responses to stress that lead to prolonged physiological activation and stress related health problems (Verkuil et al., 2011).

The social processes domain includes affiliation and attachment, social communication, self-perception and understanding, and other-perception and understanding. This system can go wrong when do not experience safety as part of a group. This is similar to the generalized unsafety theory of stress discussed in the negative valence systems, with compromised social contexts, isolation and loneliness, low social status and minority standing, unpredictable social contexts, and early negative learning all priming us to experience chronic stress (Bowlby, 1980; Baumeister and Leary, 1995; Brosschot et al., 2018; Hogan and Sherman, 2020; Van den Bergh et al., 2021).

### RDoC arousal and sensorimotor systems

The arousal and sensorimotor systems are in a sense broader level systems that encompass the other systems (Cuthbert and Kozak, 2013; Kozak and Cuthbert, 2016). Arousal involves sensitivity to external and internal stimuli and facilitating interaction with the environment according to context, which can be impacted by anxiety and depression (Lang et al., 2016; Olbrich et al., 2016; Zambrano-Vazquez et al., 2017). Circadian rhythms play an important role in homeostasis, along with the sleep/wakefulness cycle which plays an important restorative role (Gunzler et al., 2020; Pingeton et al., 2023). This system can go wrong when homeostasis is disrupted due to chronic stress or lack of quality sleep over time. The sensorimotor system involves control and execution of motor behaviors, engaging plans, and matching internal and external constraints to achieve goals. It also involves our sense of agency, the sense that we are initiating, executing and in control of our actions, the sense that our body belongs to ourselves, and general self-awareness. This system can also go wrong during prolonged chronic stress, particularly when early life adversity disrupts normal development, leading to a disrupted sense of self (Brosschot et al., 2018; Van den Bergh et al., 2021).

## Integrating current psychophysiological research findings into stress interventions

The RDoC approach provides a promising framework for integrating current psychophysiological research findings into stress interventions. Being able to assess stress across the six domains at multiple levels of functioning from chemical messengers such as cortisol to measures of social factors in health allows a thorough evaluation to be conducted. Drawing from a RDoC guided stress evaluation, stress interventions can be tailored specifically to the needs of the individual. Perhaps most importantly, the thorough assessment helps to ensure that key aspects of stress and health have been covered and this information is carefully integrated into the stress intervention to enhance the adaptative and allostatic abilities of the person.

Compassion Focused Therapy (CFT) is an excellent example of how psychophysiological research and psychophysiological interventions can be integrated with psychotherapy and stress management using the RDoC as an integrative framework. The foundational principles of CFT emphasize how negative emotion and stress occur when people lack self-compassion and interventions focus on activating positive emotions and engagement with the self and others to decrease stress. CFT also emphasizes mindful breathing exercises to directly impact heart rate variability and stress and encourages the measurement of heart rate variability as a part of the intervention process (Steffen et al., 2021). The RDoC framework provides an excellent framework for conceptualizing and integrating this information. The negative valence system aligns with the work on negative emotion and the positive valence system aligns with the work on building positive emotions. The cognitive system aligns with addressing how people think about themselves and the social system aligns with working on compassionate relationships. Finally, the arousal and modulatory systems are impacted by engaging in exercises that affect heart rate variability. Using the RDoC framework can also help in the design of research studies, focusing on measuring each of the domains in reliable and valid ways.

General approaches that can be integrated into psychotherapy or stress management interventions using the RDoC framework include heart rate variability (HRV) biofeedback and mindfulness (Steffen et al., 2017). Both modalities effectively reduce stress and have been integrated into basic stress management interventions as well as psychotherapy, and both fit well within an RDoC framework because they directly impact the negative and positive valence systems as well as the arousal and modulatory system (Khoury et al., 2015; Steffen et al., 2022). And both modalities are frequently used to collect psychophysiological data in order to understand the stress response to help guide stress interventions, as well as being a part of the intervention itself. HRV is a psychophysiological measure of particular interest in assessing both mental and physical health. HRV, a measure of the change in time intervals between heart beats, is considered a measure of positive adaptability and health. Higher HRV is associated with better adaptability and health and lower HRV is associated with worse adaptability and health. Because both HRV biofeedback and mindfulness effectively increase HRV they are being used more frequently in stress interventions (Steffen et al., 2017; Steffen and Bartlett, 2022).

### Biofeedback

Biofeedback is an interactive process where people are taught how to become more aware of and then alter their physiological activity (Lehrer et al., 2007). Sensors are attached that allow a persons' physiological signals (heart rate, respiration, etc.) to be measured in real time and shown to the person on a computer screen, allowing them to see the impact of their practice on their physiology in real time. People are also given homework assignments where they practice physiological exercises learned in session, such as breathing, in order to develop the skill more fully. A key goal of biofeedback is to help people learn to self-regulate their physiological responses, especially during times of stress.

HRV biofeedback has been shown to be particularly effective at regulating the stress response (Lehrer et al., 2007). HRV biofeedback involves measuring heart rate and respiration and showing the combined information to the client on a computer screen (Lehrer et al., 2013). People are taught to breathe at their personal resonance frequency rate, typically between 4.5 and 7 breaths per minute. Breathing instructions emphasize breathing “low and slow”, breathing low into the diaphragm and slowing the rate of breathing to the resonance frequency rate. Using feedback from the computer screen, people learn to recognize when they are in resonance, and then practice spending more time at their personal resonance frequency breathing rate. Synchronizing heart rate and breathing increases breathing efficiency and significantly increases HRV and parasympathetic activity (Lehrer and Gevirtz, 2014; Caldwell and Steffen, 2018). HRV biofeedback helps people learn to self-regulate by being more aware of their physiological stress response and using that awareness to cope better with their stress.

People also practice resonance frequency breathing at home, learning how to be in resonance without the aid of a computer screen, developing better breathing habits. Training sessions only require 15–20 min of time and are not difficult. A number of smart phone apps have been developed as well as portable biofeedback devices which makes home practice easy and effective. Because biofeedback involves developing new habits, regular practice is required to achieve full benefit. In addition to using HRV as part of an intervention strategy, HRV data is collected over time to assess change in baseline HRV as people practice and improve in breathing. HRV biofeedback is also frequently used in combination with mindfulness practices, progressive muscle relaxation, and autogenic training (Lehrer et al., 2007; Khazan, 2013). Autogenic training has also been shown to improve heart rate variability as a standalone intervention (Miu et al., 2009; Lim and Kim, 2014; Stanton et al., 2018).

### Mindfulness

As with HRV biofeedback, mindfulness also often begins with a breathing focus. Mindfulness consists of sustained moment-to-moment awareness in an open and non-judgmental way, accepting whatever one is experiencing (Grossman, 2010). Most people are not typically aware of their immediate experience, with many different thoughts occurring in the mind at any given moment, and with many of those thoughts being about the past or the future and not present focused. A key assumption of mindfulness practice is that anyone can learn to become more mindful and more present focused. Because mindfulness develops over time, it is recommended that people practice most days (if not daily), with beginners spending about 20 min each day.

There are a number of different ways to approach mindfulness, with breath focus being just one, but it is focused on here for ease of presentation. Mindfulness of breath exercises are relatively easy and straightforward to practice. People can practice in groups with a teacher and there are numerous audio-based exercises that are available for individual practice. Mindful breathing exercises have people focus on their breathing, just being with the breath, noticing the process of breathing. Interestingly, experienced meditators will naturally gravitate toward a resonance frequency breathing rate without consciously trying to. It appears that people naturally learn to recognize the calm state of resonance frequency breathing, spend more time in this breathing state, and derive benefits similar to HRV biofeedback, such as improved mood (negative and positive valence systems) and healthier physiological functioning (arousal and modulatory systems). Mindfulness is an effective approach to managing stress and improving overall wellbeing (Hofmann et al., 2010; Khoury et al., 2015; Steffen et al., 2020).

Using mindfulness and HRV biofeedback together can be an especially powerful approach to building an effective stress intervention, especially when integrated with general stress management and psychotherapeutic principles (Khazan, 2013). Conceptualizing this intervention in a RDoC framework, we would first do a thorough assessment. Each of the domains of negative and positive valence systems, cognitive and social systems, and arousal and sensorimotor systems, are reviewed within current environmental contexts and historical development. The RDoC framework recommends using a multi-modal approach to collecting data, from the very basic level such as chemical messengers, to the broadest levels of social context. To use this multi-modal approach, the assessment could begin with an interview and self-report questionnaires to examine stress, wellbeing, and social functioning. Physiological data can be collected during biofeedback on heart rate and respiration. Additionally, cortisol can be collected via saliva. Ideally, this data will be collected at home as well as in the clinic at the beginning of the intervention, during the intervention, and at the end of the intervention. This thorough data assessment will allow change and progress to be examined and can be used as feedback for intervention participants to better understand their personal stress response, realize what gains they have made, and where to best focus their efforts in the future.

## Conclusions

The RDoC is a promising framework for integrating psychophysiological research into psychotherapy and stress management interventions. The RDoC framework is a dimensional, multi-modal approach that allows for integration of information from multiple levels of functioning in the context of environments and development. Integrating psychophysiological research on stress using the RDoC framework can direct and amplify stress management and psychotherapeutic interventions. The RDoC provides a clear foundation for conceptualizing the stress response in terms of important concepts such as allostasis and adaptation. Psychophysiological approaches can be used in the context of modalities such as biofeedback, Autogenics, and mindfulness to collect psychophysiological data that can then be integrated into a broader therapeutic framework for conducting stress interventions. Overall, the RDoC provides a strong framework to assess and integrate physiological and psychological data and thereby improve stress interventions.

## Data availability statement

The original contributions presented in the study are included in the article/supplementary material, further inquiries can be directed to the corresponding author.

## Author contributions

The author confirms being the sole contributor of this work and has approved it for publication.
